# Gut metagenomic analysis of gastric cancer patients reveals *Akkermansia*, *Gammaproteobacteria*, and *Veillonella* microbiota as potential non-invasive biomarkers

**DOI:** 10.1186/s44342-024-00001-8

**Published:** 2024-05-21

**Authors:** Anju R. Nath, Jeyakumar Natarajan

**Affiliations:** Data Mining and Text Mining Laboratory, Department of Bioinformatics, Bharathiar University, Coimbatore, 641 046 India

**Keywords:** Gastric cancer, Metagenomic analysis, Gut microbiota, Diversity analysis, QIIME2

## Abstract

The goal of the study was to investigate the changes in the gut microbiota during the advancement of gastric cancer (GC) and identify pertinent taxa associated with the disease. We used a public fecal amplicon gastric cancer dataset from the Sequence Retrieval Archive (SRA), of patients with GC, gastritis, and healthy individuals. We did sequence pre-processing, including quality filtering of the sequences. Then, we performed a diversity analysis, evaluating α- and β-diversity. Next, taxonomic composition analysis was performed and the relative abundances of different taxa at the phylum and genus levels were compared between GC, gastritis, and healthy controls. The obtained results were subsequently subjected to statistical validation. To conclude, metagenomic function prediction was carried out, followed by correlation analysis between the microbiota and KEGG pathways. α analysis revealed a significant difference between male and female categories, while β analysis demonstrated significant distinctions between GC, gastritis, and healthy controls, as well as between sexes within the GC and gastritis groups. The statistically confirmed taxonomic composition analysis highlighted the presence of the microbes *Bacteroides* and *Veillonella*. Furthermore, through metagenomic prediction analysis and correlation analysis with pathways, three taxa, namely *Akkermansia*, *Gammaproteobacteria*, and *Veillonella*, were identified as potential biomarkers for GC. Additionally, this study reports, for the first time, the presence of two bacteria, *Desulfobacteriota* and *Synergistota*, in GC, necessitating further investigation. Overall, this research sheds light on the potential involvement of gut microbiota in GC pathophysiology; however, additional studies are warranted to explore its functional significance.

**Supplementary Information:**

The online version contains supplementary material available at 10.1186/s44342-024-00001-8.

## Introduction

Gastric cancer (GC) is one of the most frequent and fatal cancers worldwide. It is the third most common cause of cancer-related deaths worldwide, according to GLOBOCAN 2018 data [1]. Hereditary, environmental, lifestyle, and microbes have a role in the development of GC [2]. *Helicobacter pylori* infection is often regarded as the most important risk factor for the development of GC [3]. Furthermore, several studies based on high-throughput sequencing technologies have revealed that changes in the microbiota of the stomach, other than *H. pylori*, such as *Streptococcus*, *Lactococcus*, *Veillonella*, and *Fusobacteriaceae*, are associated with the development of gastric cancer [4, 5].

Microbial metabolites serve as crucial mediators in mammalian symbiotic connections with microbial cells in the gastrointestinal tract. In recent decades, the gut microbiota has received much interest, leading to the discovery of many new areas in which bacterial transformation could play a key role in human health and disease. Cancers of the gastrointestinal system, such as gastric and colorectal cancers, are associated with the gut microbiota [6]. However, little is known about the characteristics of gut microbiota composition that are associated with gastric cancer [7]. According to previous studies, changes in the gastric flora may cause a chronic inflammatory response, which can alter the gastric carcinogenesis process [8]. Gut microorganisms found in tumor tissues and feces may be used as a prognostic, diagnostic, and therapeutic marker for GC. Wu et al. proposed the use of the *Streptococcus* and *Veillonella* genus as GC biomarkers in their study [9].

In this study, we investigated changes in the gut microbiota during the progression of GC to identify the most relevant taxa associated with the disease and assess the potential of the microbiota as a diagnostic biomarker for GC. A diversity analysis was performed, followed by taxonomic composition analysis and metagenomic function prediction.

## Materials and methods

### Public metagenomic cohorts of patients with gastric cancer, gastritis, and controls

We used the public fecal amplicon gastric cancer dataset (Bioproject-PRJNA639644) from SRA (Sequence Retrieval Archive), which included 83 patients with gastric cancer, 54 patients with gastritis, and 61 healthy people (Table 1). The dataset comprised of 116 males and 82 females. In the previous study by Zhang et al. (Bioproject-PRJNA639644), the DNA was extracted from stool samples and the PCR amplification of bacterial 16S rRNA genes V4 region was done using Illumina HiSeq4000 platform [7].

**Table 1:** Dataset used in the study

	Gastric cancer	Gastritis	Healthy
Female	27	27	28
Male	56	27	33

### Sequence pre-processing

Fecal metagenomic amplicon sequences were subjected to preprocessing. The FASTQC tool was used to assess the quality of the sequence [10]. The sequences were then quality filtered in the Quantitative Insights into Microbial Ecology (QIIME2) pipeline [11] using the Deblur tool [12]. Sequences having a length of > 150 bp and with Phred score > 20 were retained. QIIME2 is a sophisticated microbiome analysis tool that emphasizes data and analysis transparency. The sequence quality parameters were set to a minimum Phred score of 25. And sequences having a length of > 150 bp were retained.

### Biological diversity assessments

A diversity index is a quantitative measure of how many types of families and species exist in a community [13]. Biological diversity was assessed using the Qiime2 tool [11]. Here, α- diversity was used to measure species diversity within a community. For α-diversity analysis, Shannon’s diversity index and Faith’s phylogenetic diversity were used. Shannon’s diversity index is a quantitative assessment of community richness. The qualitative metric of community richness was determined by Faith’s phylogenetic diversity. However, Faith’s phylogenetic diversity considers both the feature’s phylogenetic relationship and the diversity of the community. Furthermore, β-diversity analysis was used to calculate the species diversity between the communities. For β-diversity analysis, Unweighted UniFrac distance and Weighted UniFrac distance were used [14]. The qualitative measure of community dissimilarity was given by the Unweighted UniFrac distance. A quantitative measure of community dissimilarity was provided by the weighted UniFrac distance. Unweighted and weighted UniFarc distances, on the other hand, consider evolutionary relationships between features as well as community dissimilarity [14]. Further, the results were confirmed with the PERMANOVA test. PERMANOVA test is frequently conducted following β-diversity analysis to determine whether there are statistically significant differences in the total multivariate composition of samples among groups or conditions. While β-diversity analysis aids in exploring and visualizing the dissimilarity patterns in the data, PERMANOVA offers a formal statistical test to evaluate whether these patterns are significant and can be linked to the grouping factor of interest. These studies collectively offer a thorough grasp of the multivariate composition and its connection to the variables being studied.

### Taxonomic composition analysis

The QIIME2 tool was used to explore the taxonomic composition of the samples [11]. The identity and abundance of species or taxonomic groupings within a site or body are referred to as the taxonomic composition. In this study, we used the SILVA-138-99-nb-classifier to map taxonomy. To visualize taxa abundance, we used the R package Microeco [15].

### Comparison of taxonomic composition

LEfSe (Linear discriminant analytic Effect Size) is a statistical method that is used to find characteristics (such as biomarkers, taxa, or genes) that are differently prevalent between various groups or situations [16]. To identify possibly biologically significant differences, LEfSe focuses on finding features that not only demonstrate statistically significant differences but also have large effect sizes. To identify the characteristics most likely to account for differences across groups, LEfSe employs linear discriminant analysis (LDA). To find features with significantly differing abundances across groups, it first runs a non-parametric Kruskal-Walli’s rank sum test. The next step is to estimate the effect size of each differentially abundant feature using LDA, and the statistical significance of the effect size is evaluated using a resampling-based method. The abundance differences with an LDA score > 3.0 were considered statistically significant.

Along with LEfSe, we performed a multi-factor analysis with MicrobiomeAnalyst, which allowed us to investigate the concurrent influence of several factors on microbial composition [17]. Taking into consideration any confounding factors, multi-factor analysis aids in revealing substantial correlations between microbial taxa and the conditions of interest. To account for its effects and especially look at the independent influence of the circumstances on the microbiome, we also included sex as a covariate. Results with FDR < 0.05 were considered significant.

### Prediction of metagenomic functions

Tax4Fun2 was used to make functional predictions [18]. Tax4Fun2 uses 16S rRNA gene sequencing data to predict and explore functional profiles of bacterial communities. BLASTp with R package diamond [19] is used to create functional profiles against the KEGG KO database.

### Correlation analysis using Spearman to identify taxa that contribute to the functional abundance

Using the taxonomic abundance profiles, we performed an FDR-corrected Spearman test to correlate the relative abundance of each genus with the paired relative abundance of each pathway across samples. Significant correlations were identified as those with FDR < 0.05 and |*ρ*|< 0.9. Highly correlated features were considered dependent features and excluded from this analysis (correlation coefficient > 0.9).

## Results

### Diversity estimates of the microbiota

In this study, a total of 198 publicly available metagenome sequencing datasets were included and processed, comprising 83 samples from patients with GC, 54 samples from patients with gastritis, and 61 samples from healthy individuals. The differences in microbiota in metadata features such as disease and sex were measured by the α and β diversities, and the α diversity of the samples was analyzed using Shannon and Faith’s phylogenetic diversity (PD) indices. The 16S fecal microbiota was first analyzed in GC, gastritis, and healthy controls. However, there were no significant differences in the α diversity analysis by both Shannon and Faith’s PD (*p* = 0.495 and 0.150, respectively) (Table 2). Next, the α analysis of males and females from the three categories (GC, gastritis, and healthy) was performed. Significant differences were observed between males and females in the GC category (*p* = 0.004 and *p* = 0.0003, respectively) (Supplementary Fig. 1) implying species richness within the samples. However, there were no differences between the males and females in the gastritis and healthy categories.

**Table 2:** α-diversity pair-wise values of Shannon and Faith’s PD indices for GC, gastritis, and healthy control

**Group 1**	**Group 2**	**Shannon index** ***p*-value**	**Faith’s PD index** ***p*-value**
Gastric cancer	Gastritis	0.445	0.893
Gastric cancer	Health	0.642	0.070
Gastritis	Health	0.216	0.158

β-diversity analyses, performed using unweighted and weighted UniFrac algorithms, performed on GC, gastritis, and healthy controls, showed separation between the three categories (*p* = 0.001 and 0.014, respectively) (Supplementary Figs. 2 and 3). This result was confirmed by PERMANOVA tests, applied on both unweighted and weighted distant matrices (Table 3), performed separately on GC vs. gastritis (*p* = 0.125 and *p* = 0.111, respectively), GC vs. health (*p* = 0.015 for both analyses), and gastritis vs. health (*p* = 0.003 and *p* = 0.404, respectively). A significant difference was observed between sex in GC and gastritis categories in unweighted analysis (*p* = 0.001 and *p* = 0.045, respectively) (Supplementary Fig. 4). But weighted UniFarc analysis showed no significance.

**Table 3:** Pairwise PERMANOVA results for GC, gastritis, and healthy control (significant values in bold)

**Group 1**	**Group 2**	**Unweighted UniFarc distance**	**Weighted UniFarc distance**
		***p*-value**	***p*-value**
Gastric cancer	Gastritis	0.125	0.111
Gastric cancer	Health	**0.010**	**0.015**
Gastritis	Health	**0.001**	0.404

### QIIME2-based taxonomic composition analysis

**Fig. 1:**
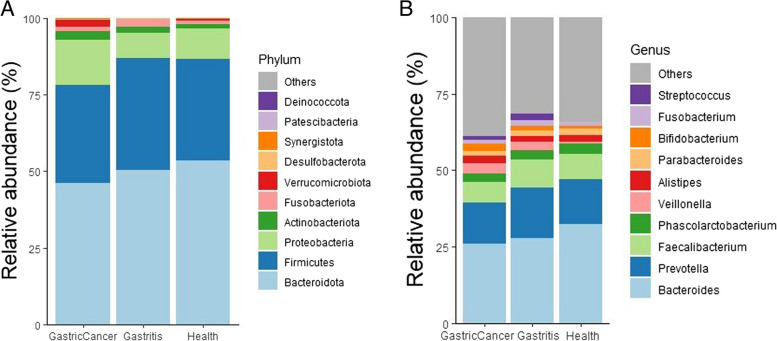
Bar plot showing the relative abundance of the microbiota at **a** phylum level and **b** genus level, in gastric cancer, gastritis, and healthy individuals

We observed that patients with GC had a higher relative abundance of *Proteobacteria*, *Actinobacteria*, *Verrucomicrobiota*, *Desulfobacteriota*, and *Synergistota* at the phylum level compared to patients with gastritis and healthy individuals. Previous studies have reported that *Proteobacteria*, *Verrucomicrobiota*, and *Actinobacteria* are associated with GC risk [20, 21]. However, the presence of *Desulfobacteriota* and *Synergistota* in GC has not been previously investigated, making our study the first to report on these microorganisms in relation to GC. Additionally, we found that *Firmicutes* and *Fusobacteriota* were more abundant in patients with gastritis than in those with GC and healthy individuals, while *Bacteriodota* exhibited higher levels in healthy individuals compared to both GC and gastritis patients. When comparing GC patients with gastritis and healthy subjects, *Bacteriodota* showed lower abundance in GC patients (Fig. 1a) (Supplementary Table 1).

At the genus level, we observed higher relative abundances of *Bifidobacterium*, *Streptococcus*, and *Veillonella* in patients with GC compared to those with gastritis and healthy controls. Furthermore, the relative abundances of *Bacteroides*, *Faecalibacterium*, and *Prevotella* were lower in GC patients compared to gastritis patients and healthy controls. However, some studies have reported contradictory results regarding *Bifidobacterium longum*, which has been suggested to have anti-angiogenic and anti-proliferative properties in GC [22]. The occurrence of GC was substantially linked to changes in *Streptococcus* [23], and the presence of *Veillonella* in GC has been reported previously [24]. In gastritis, higher levels of *Fusobacterium*, *Prevotella*, and *Streptococcus* were observed compared to GC and healthy controls. The abundance of *Bacteroides*, *Phascolarctobacterium*, and *Parabacteroides* was higher in healthy controls than in patients with GC and gastritis, while *Veillonella* levels were lower in the healthy group compared to the other two groups (Fig. 1b).

**Fig. 2:**
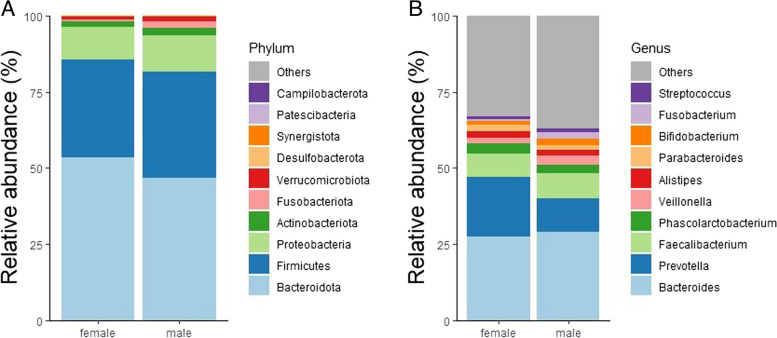
Bar plot showing the relative abundance of the microbiota at **a** phylum level and **b** genus level in males and females

Regarding the phylum-level analysis in terms of sex, we found that *Firmicutes*, *Proteobacteria*, *Fusobacteria*, and *Verrucomicrobiota* were more abundant in males (Fig. 2a). Previous studies have also reported that *Firmicutes*, *Proteobacteria*, *Fusobacteria*, and *Verrucomicrobiota* are associated with GC risk [20]. It is well-established that the prevalence of GC is higher in males than in females [1, 25]. The specific role of these microbes in male GC requires further study. In females, at the genus level, we observed higher abundances of *Prevotella* and *Parabacteroides*, while *Bacteroides*, *Fusobacterium*, *Veillonella*, and *Bifidobacterium* showed lower abundances (Fig. 2b).

### Comparison of taxonomic composition by LEfSe for 16S rRNA-based metagenomic biomarker and multi-factor analysis

The differential abundance test is a critical component of the analysis of microbial community data. It can be used to identify significant taxa when determining the community differences between groups. Linear discriminant analysis effect size (LEfSe) is a tool developed by the Huttenhower group that uses relative abundances to identify biomarkers between two or more groups [16].

**Fig. 3:**
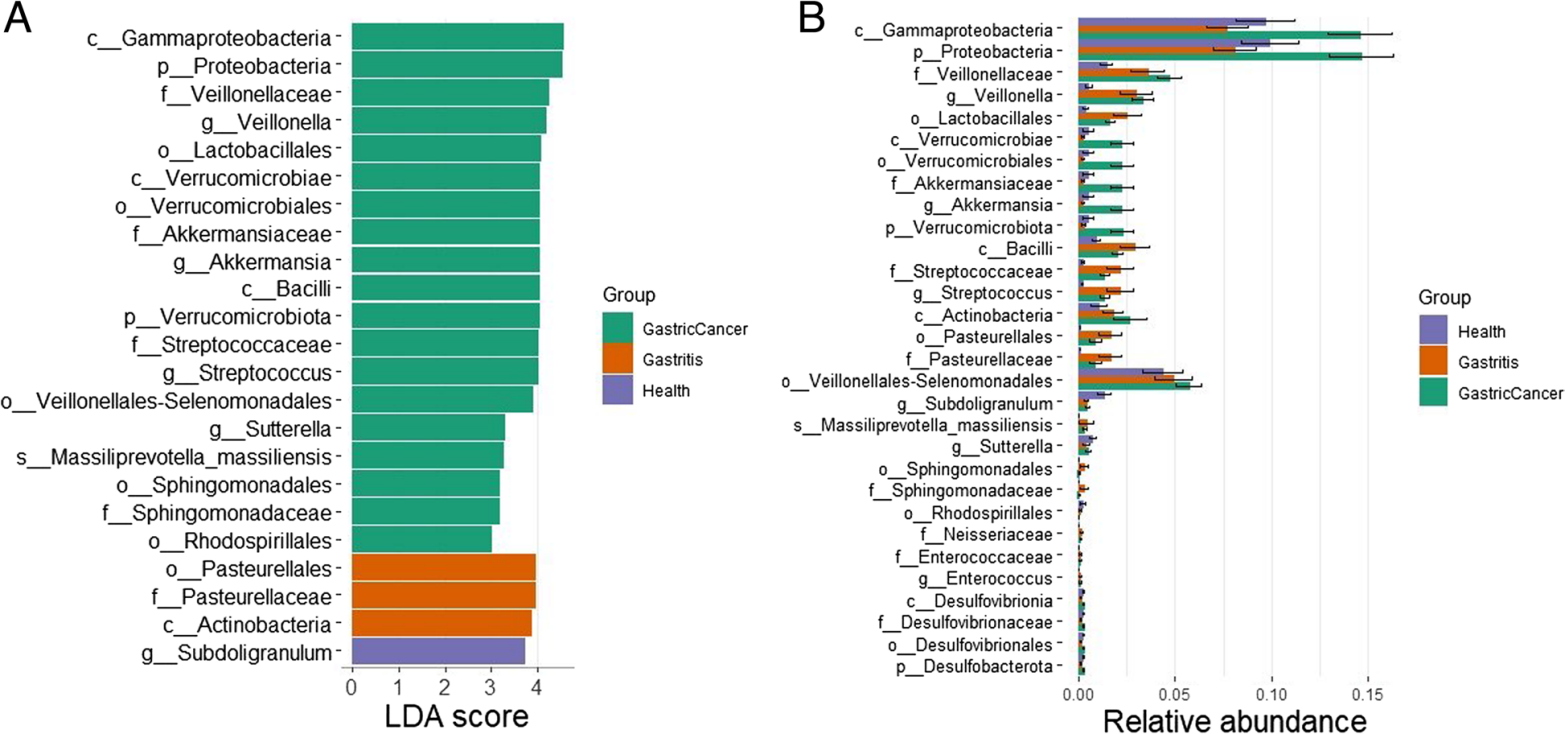
LEfSe analysis identified the most differentially abundant taxa in gastric cancer, gastritis, and healthy controls. **A** Histogram of the linear discriminant analysis (LDA) scores (minimum score = 3). **B** Plot showing the abundance of biomarkers identified by LEfSe

*c_Gammaproteobacteria*, *Veillonella*, *Akkermansia*, and *Verrucomicrobiota* were identified as potential GC biomarkers. *Gammaproteobacteria* and *Proteobacteria* were found to be significantly abundant in patients with GC [26, 27] (Fig. 3). Guo et al. discovered *Veillonella* in GC patients with an advanced gastric lesion [24]. There was a clear gradation of *Veillonella* from healthy individuals to those with GC through gastritis. A perioperative study on patients during perioperative study suggests that the abundance of *Akkermansia* in postoperative samples was enriched when compared to GC samples, which contradicts our findings [21]. Although *Akkermansia* is considered a gut-friendly microbe, its association with different factors may make it pathogenic [28]. Another study on colorectal cancer by Osman et al. showed the over-representation of *Akkermansia* in cancer samples [29]. *Pasteurellaceace* and *Actinobacteria* were identified in gastritis and *Subdoligranulum* was identified in healthy controls.

**Fig. 4:**
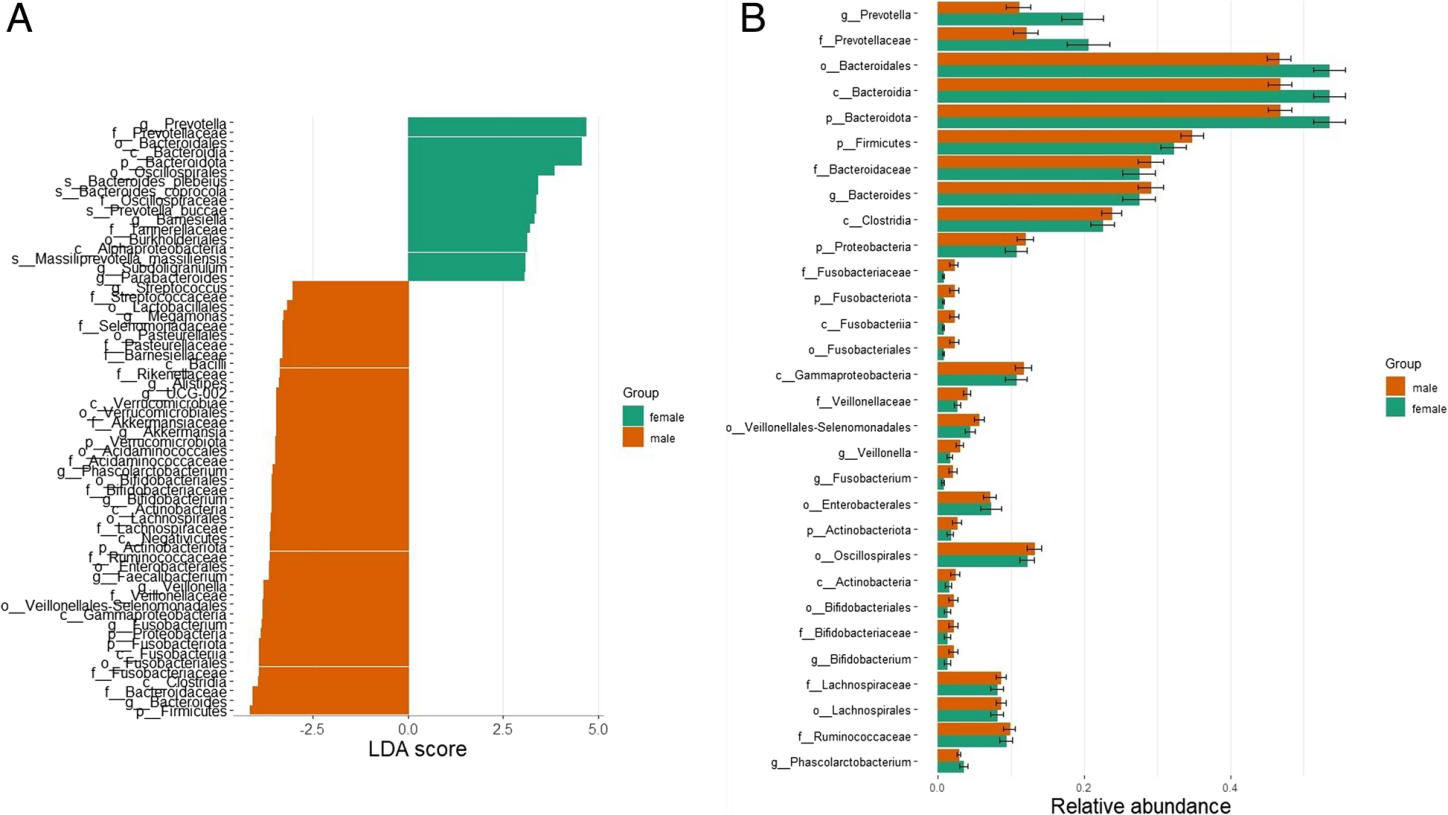
LEfSe analysis identified the most differentially abundant taxa in males and females. **A** Histogram of the linear discriminant analysis (LDA) scores (minimum score = 3). **B** Plot showing the abundance of biomarkers identified by LEfSe

We identified *Prevotella* and *Bacteriodota* as potential biomarkers in females. *Firmicutes*, *Bacteroides*, *Proteobacteria*, *Clostridia*, *Veillonella*, *Fusobacterium*, and *Gammaproteobacteria* were identified as potential biomarkers in males (Fig. 4).

**Fig. 5:**
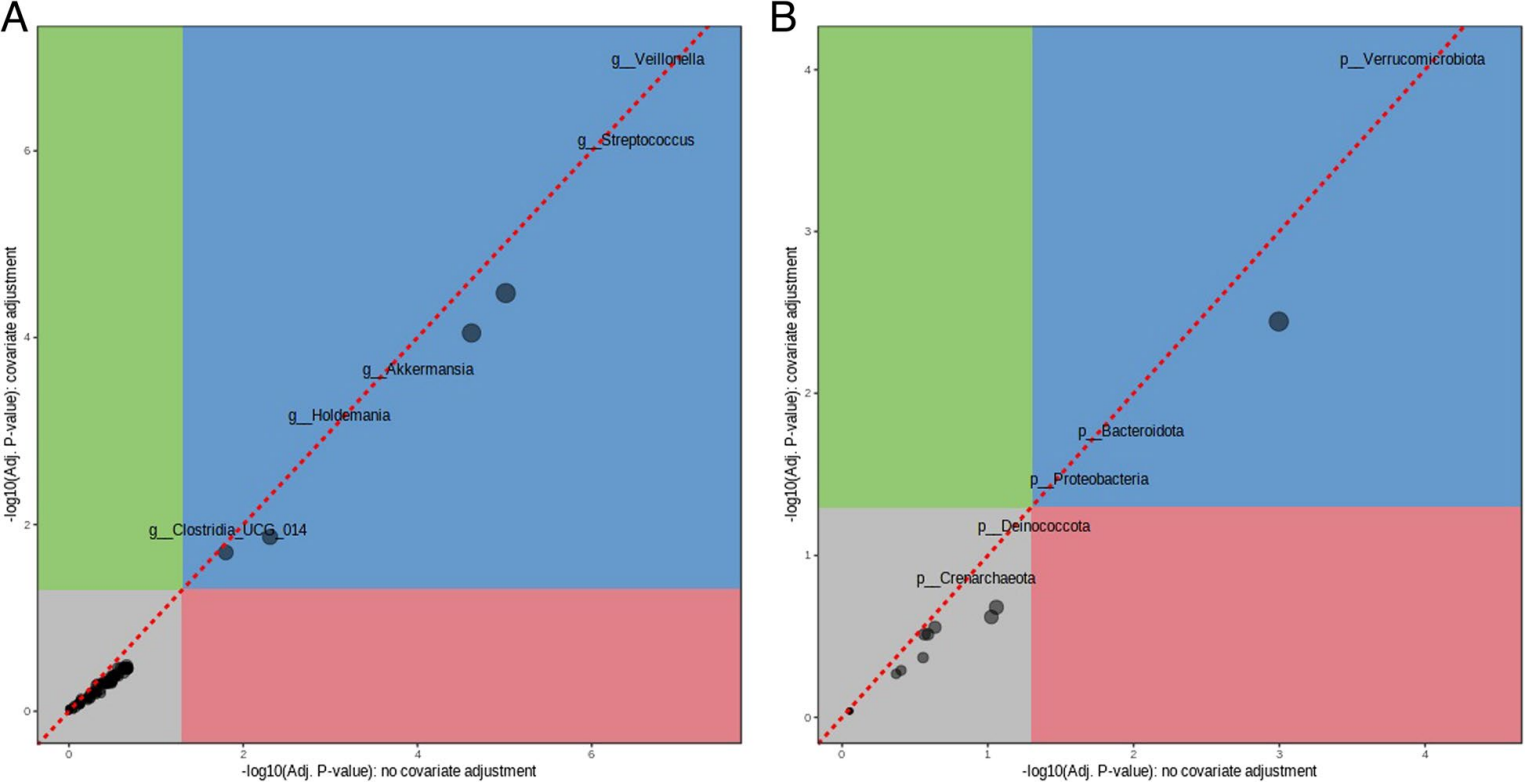
Multi-factor analysis showing covariate plot between GC and healthy individuals in **A** genus level and **B** phylum level

After performing LEfSe analysis, we carried out a multi-factor analysis and included sex as a covariate to take into consideration its potential impact on the microbial composition. We discovered that in the genus level, three microbes, *Akkermansia, Streptococcus, and Veillonella* were significant when compared to healthy individuals, indicating their probable role in GC (Fig. 5A) and in phylum level, *Verrucomicrobiota* was significantly higher in GC (Fig. 5B).

**Fig. 6:**
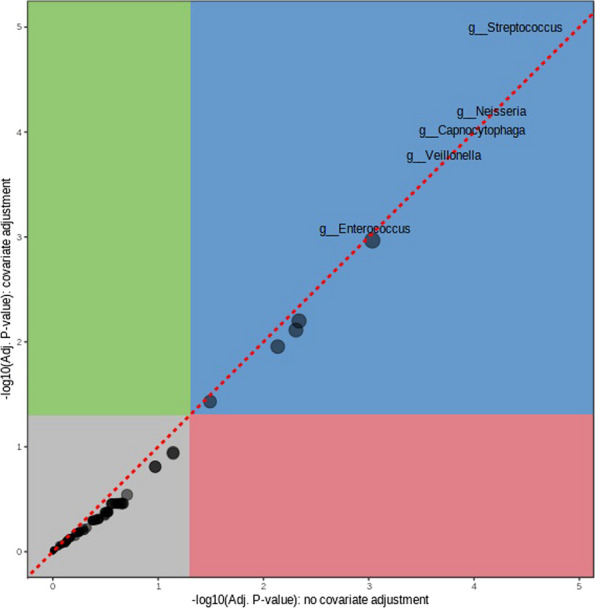
Multi-factor analysis showing covariate plot between gastritis and healthy individuals in phylum level

In the analysis between gastritis and healthy individuals at the genus level, four microbes, *Enterococcus*, *Capnocytophaga*, *Neisseria*, *Streptococcus*, and *Veillonella* were significantly higher in gastritis (Fig. 6). This implies that these genera might be involved in gastritis and could contribute to the microbial dysbiosis associated with gastritis.

### Metagenomic functional prediction analysis

**Fig. 7:**
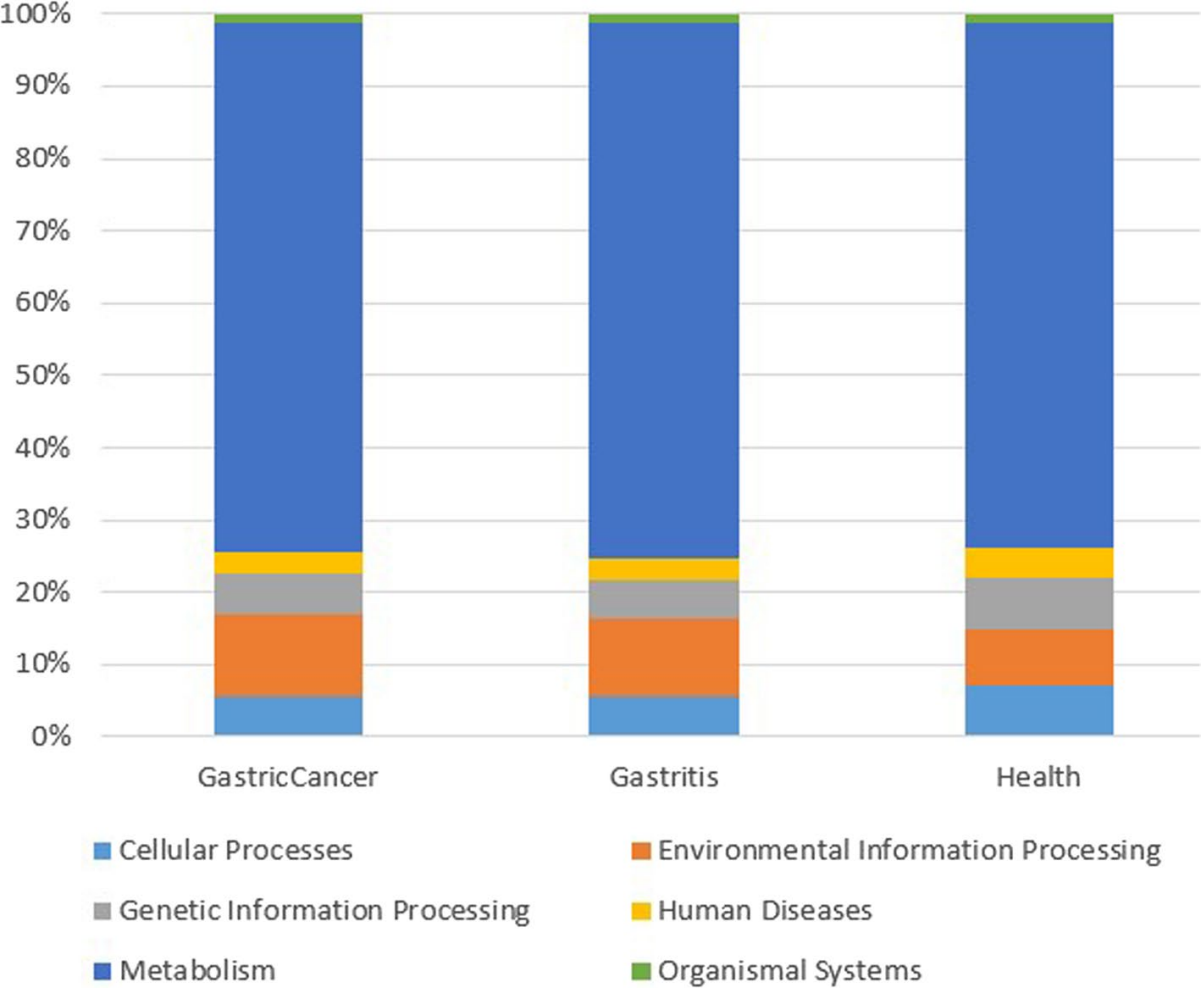
Bar plot showing the level 1 KEGG pathways enriched in GC, gastritis, and healthy individuals

We performed a functional prediction of the bacterial communities using Tax4Fun2. A total of 334 pathways were identified in the six KEGG level 1 groups: metabolism, human diseases, organismal system, cellular process, environmental information processing, and genetic information processing. Metagenomic predicted functions showed that “Metabolism” had the highest proportion of genetic sequences involved in all three categories (Fig. 7).

In both GC and gastritis “environmental information processing” was the second highest. But in healthy individuals, “cellular process”, “genetic information processing”, and “human disease” were the highest.

**Fig. 8:**
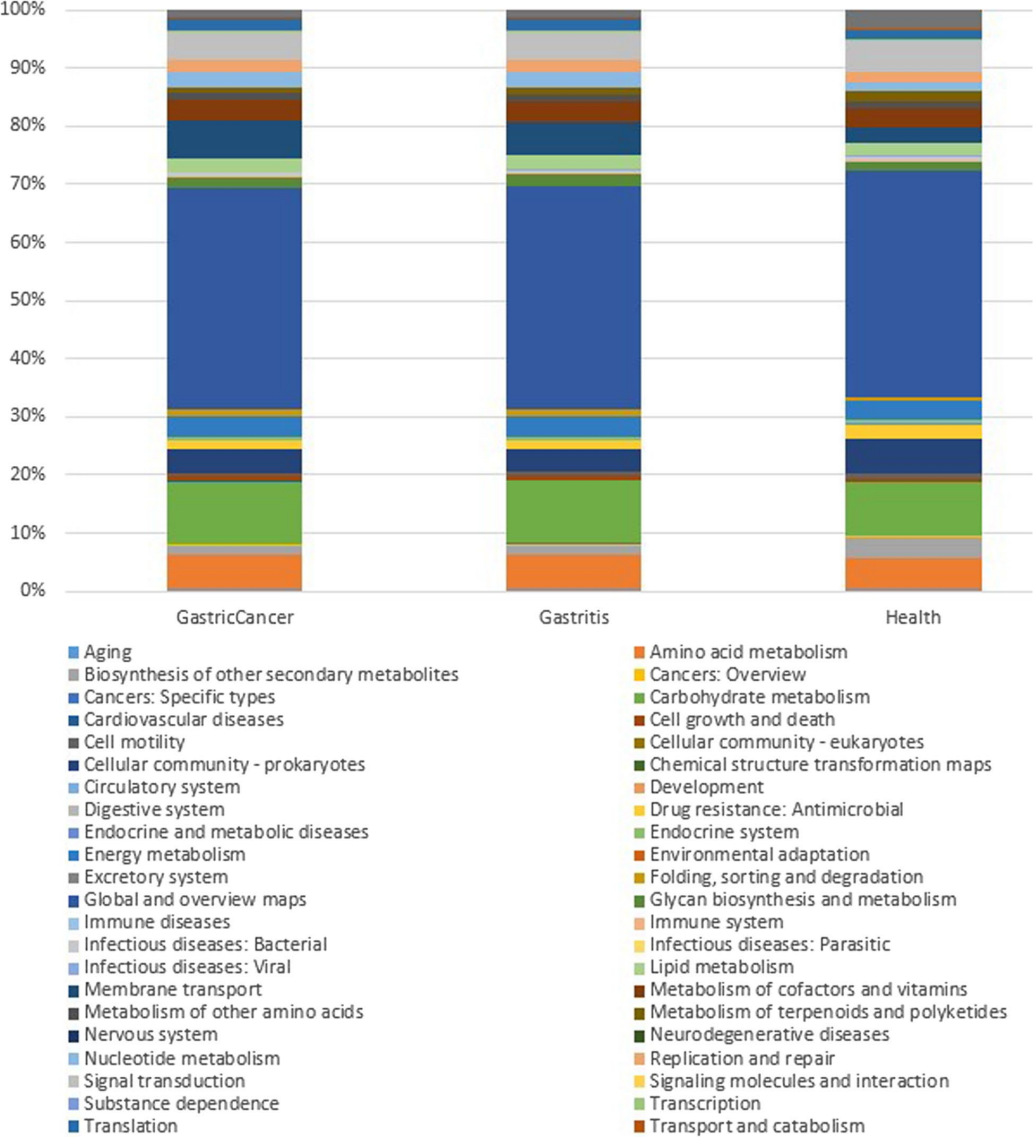
Bar plot showing the level 2 KEGG pathways enriched in GC, gastritis, and healthy individuals

At level 2, 46 pathways were identified. At this level, “global and overview maps” were highest in proportion in all three categories, GC, gastritis, and healthy control (Fig. 8). In both GC and gastritis, the metabolism of amino acids, carbohydrates, cofactors, and vitamins is the same.

Next, we performed a correlation analysis between the microbiota and KEGG pathways using the FDR-corrected Spearman correlation analysis (FDR < 0.05). The significantly enriched microbiota in GC was used to analyze the correlation with the pathways. Pathways showing the strongest correlation were considered for further analysis.

*p_Proteobacteria* and *c_Gammaproteobacteria* were having a positive correlation with the pathway, “infectious disease, bacterial – bacterial invasion of epithelial cells” (|*ρ*|= 0.86536) and xenobiotics biodegradation and metabolism (|*ρ*|= 0.826327). A study by Litvak et al. showed that the dysbiotic growth of *Proteobacteria* is a putative diagnostic microbial marker of epithelial dysfunction [30]. The epithelial tissue is the most typical location for the emergence of malignancies. A xenobiotic is a chemical compound detected in an organism that has not been naturally created. Even though studies in humans relating to the correlation of *Gammaproteobacteria* with xenobiotic biodegradation are not available, a study by Madueño et al. in freshwater sediments suggests that the *Gammaproteobacteria* has a role in degrading xenobiotic compounds [31]. Next, *Verrucomicrobiota* and genus *Akkermansia* were positively correlated with the pathways, Lipid metabolism (|*ρ*|= 0.869666) followed by Metabolism of terpenoids and polyketides (|*ρ*|= 0.686225). Studies in mice have proved that *Akkermansia* robustly correlates with lipid metabolism [32]. Alterations in lipid metabolism are widely acknowledged as a characteristic of cancer cells. Oncogenic signals directly control alterations in lipid-metabolizing enzyme expression and activity [33]. Although studies related to the metabolism of terpenoids and polyketides with GC are not available, Su et al. showed the role of microbial genes related to the metabolism of terpenoids and polyketides in oral cancer [34]. *Veillonella dispar* is a *Veillonella* species that participates in nitrogen metabolism, which is a type of energy metabolism [35]. Nitrogen metabolism plays an important role in proliferating cancer cells [36]; however, studies linking *Veillonella* to the pathway of metabolism of cofactors and vitamins are not available.

## Discussions

In assessing alpha diversity, which offers insights into species richness within individual samples, Shannon, and Faith’s phylogenetic diversity (PD) indices were employed. Surprisingly, no significant differences were observed in alpha diversity among samples from individuals with GC, gastritis, and healthy controls. This finding implies that, at the taxonomic level investigated in this study, the overall richness and evenness of the gut microbial communities were comparable across these three conditions. Upon further analysis, differentiating by gender revealed intriguing patterns. In the GC category, a significant divergence in alpha diversity was evident between males and females, indicating variations in species richness within the microbial communities. In contrast, no such distinctions were observed in the gastritis and healthy control categories.

The analysis of beta diversity, which assesses variations in the microbial composition of samples, demonstrated significant differences between GC patients, gastritis patients, and healthy controls. Significant separations between these three categories were shown by both unweighted and weighted UniFrac analyses, highlighting the presence of disease-specific microbial signatures. These results were further corroborated by the pairwise PERMANOVA results, which showed statistically significant differences in microbial composition between GC and gastritis, GC and health, and gastritis and health. Interestingly, the unweighted UniFrac analysis also identified significant differences between sexes in the GC and gastritis categories, suggesting a potential influence of gender on the gut microbial community structure in these disease states.

The findings of our study show that patients with GC, gastritis, and healthy controls have significantly different gut microbiota compositions. Patients with GC exhibited a well-defined microbial profile distinguished by higher relative abundances of *Proteobacteria*, *Actinobacteria*, *Verrucomicrobiota*, *Desulfobacteriota*, and *Synergistota* at the phylum level. These results support earlier studies that connected *Proteobacteria*, *Verrucomicrobiota*, and *Actinobacteria* to a higher risk of GC. We report for the first time the existence of *Desulfobacteriota* and *Synergistota* in GC, suggesting the need for more research to determine their potential contribution to the pathogenesis of GC.

We observed differences in the abundance of several taxa between GC, gastritis, and healthy controls at the genus level. In contrast to *Bacteroides*, *Faecalibacterium*, and *Prevotella*, which were shown to be less prevalent in GC patients, *Bifidobacterium*, *Streptococcus*, and *Veillonella* were found to be more common. These results are in accordance with other studies that linked *Streptococcus* and *Veillonella* to GC, even though *Bifidobacterium longum* has produced contradicting results. *Fusobacterium*, *Prevotella*, and *Streptococcus* are more prevalent in gastritis than in GC and healthy individuals, which may indicate that they are involved in the pathophysiology of the condition. The increased prevalence of *Bacteroides, Phascolarctobacterium*, and *Parabacteroides* in healthy controls may point to their importance in preserving gut homeostasis and posing a possible risk factor for GC development.

At the phylum level, the results of our investigation also showed sex-related changes in the composition of the gut microbiota. *Firmicutes*, *Proteobacteria*, *Fusobacteria*, and *Verrucomicrobiota*—all of which have been linked to GC risk—were found in larger abundances in males. These results add to our knowledge of why men have a higher prevalence of GC than women. To clarify the precise methods by which these bacteria may contribute to the establishment of male-specific GCs, more research is required.

Significant differences in microbial composition between people with GC and healthy individuals, as well as between people with gastritis and healthy individuals, indicate that microbial taxa may be involved in these diseases. The genus-level occurrence of *Veillonella*, *Streptococcus*, and *Akkermansia* in GC indicates their potential function as disease-causing agents or biomarkers. Elevated levels of *Enterococcus*, *Capnocytophaga*, *Neisseria*, *Streptococcus*, and *Veillonella* in people with gastritis provide more evidence that microbial dysbiosis contributes to the inflammatory processes linked to gastritis. These results imply that changes in the composition of these taxa may influence the occurrence or persistence of gastritis. Additionally, a wider dysregulation of this microbial community in the illness state is suggested by the greater abundance of *Verrucomicrobiota* at the phylum level in GC.

We were able to examine the relevance of microbiological differences between males and females while considering their potential confounding effects by including sex as a covariate in our analysis. To gain a deeper understanding of the microbial relationships in our investigation, we were able to isolate and analyze the independent effects of the conditions (gastric cancer, gastritis, and healthy individuals) on microbial composition by controlling for sex. Insights into the underlying mechanisms and potential therapeutic targets for gastric cancer and gastritis may be gained by more research into the functional role of these bacteria and their interactions with the host.

The correlation study between the microbiota and KEGG pathways revealed important information on potential associations between bacterial taxa and functional GC pathways. *Proteobacteria* have been positively correlated with the mechanisms for bacterial invasion of epithelial cells and xenobiotic biodegradation, suggesting that they may play a role in the progression of disease. *Proteobacteria* have been linked to dysbiotic epithelial development, which is consistent with the typical site of malignancies. Furthermore, a study in freshwater sediments suggests that *Gammaproteobacteria* have a role in the degradation of xenobiotic substances. In the context of our Spearman correlation analysis, it is crucial to acknowledge that the correlation was exclusively identified between *c_Gammaproteobacteria* and the pathways, infectious disease, bacterial–bacterial invasion of epithelial cells, and xenobiotics biodegradation and metabolism. While this provides valuable insights, it is important to recognize that the taxonomic resolution is limited, extending only to the broader taxonomic category and not to finer levels such as genera or species. The diversity within the *Gammaproteobacteria* class, encompassing more than 250 bacterial genera with diverse biological properties, poses a challenge in pinpointing the specific members contributing to the observed correlation. Therefore, our findings, while informative, should be interpreted with the understanding that the taxonomic information at a higher resolution could offer more nuanced insights into the microbial community dynamics.

The potential contributions of *Verrucomicrobiota* and *Akkermansia* to cancer-related metabolic processes are highlighted by the two organisms’ favorable associations with lipid metabolism and the metabolism of terpenoids and polyketides, respectively. In mice, *Akkermansia* has been related to changes in lipid metabolism, and cancer cells are known to exhibit these changes in lipid metabolism. Although research explicitly looking into the metabolism of terpenoids and polyketides in GC are scarce, oral cancer has been linked to microbial genes involved in these pathways.

*Veillonella dispar* takes part in nitrogen metabolism, which is essential for the energy metabolism of cancer cells that are actively multiplying. Further research is necessary, nevertheless, to determine how *Veillonella* is related to the metabolism of cofactors and vitamins.

Earlier, Zhang et al. [7] carried out how the gut microbiome changes as GC progresses. In their work, the potential of the microbiome for GC diagnosis as well as the pertinent taxa linked with GC were examined. In continuation, the present study further extends and demonstrates the role of these bacteria in various pathways and a gender-based analysis to identify the presence of microbiome in both males and females. Our findings indicated that men have higher levels of cancer-causing microorganisms than women. We have also predicted the metagenomic pathways and the relationships between bacteria and these pathways and their relevance in GC. These microbes’ pathway analysis results suggest that they might contribute to the development of GC. Additionally, two previously unreported bacteria from GC, *Desulfobacteriota* and *Synergistota*, have been reported in our investigation. Further findings include GC samples had a lot of *Akkermansia*, which is regarded as a gut-friendly bacterium. Understanding the function of *Akkermansia* in cancer from our results is a topic of interest to further investigate.

Gastric cancer is usually related to *H. Pylori* infection, and it was believed that the stomach doesn’t have microbiota other than *H. pylori* because of its acidic nature. But nowadays several studies have proved that the stomach contains microbiota other than *H. pylori* [37–39]. Also, the connection between gastric microbiota and gut microbiota has been proven through recent studies [40, 41]. A study by Sarhadi et al. stated that the patients with aggressive gastric tumor types such as diffuse adenocarcinoma have lower gut microbiota diversity [40]. In another study by Gao et al., the fecal microbiota composition was profiled, revealing significant associations with *H. pylori* infection and gastric lesion severity, particularly noting alterations in *Bacteroidetes*, *Firmicutes*, and *Proteobacteria*, offering novel insights for future investigations on *H. pylori*-related carcinogenesis and post-eradication gut microbiota changes [42]. *Veillonella* microbiota has been identified in association with both the gastric microbiome and *H. pylori* [39, 43]. In contrast, our investigation did not reveal a significant association between the gastric microbiota and *Akkermansia* or *Gammaproteobacteria*. Notably, *Veillonella* has also been linked to the salivary microbiota in GC patients [44]. These findings underscore the prominence of *Veillonella* as a substantial microbiota associated with GC, emphasizing its potential significance in the context of this disease.

The fecal microbiome was investigated in this study as it is the habitat of microbial populations that have substantial health and disease implications. When comparing GC to gastritis and health, we discovered that there were significant differences in the relative abundance of specific taxa. Subsequently, a functional analysis of metagenomic biomarkers suggested that microbes such as *Akkermansia*, *Gammaproteobacteria*, and *Veillonella* might be used as GC biomarkers. Pathway analysis of these microbes indicated that they may play a role in gastric carcinogenesis. By analyzing metagenomic biomarkers in fecal samples, our study advances the non-invasive early diagnosis of GC. Further research into the link between microorganisms and pathways could reveal the role of microbiota in the pathogenesis of gastric cancer. Moreover, these findings will aid our understanding of the pathophysiology of GC from the perspective of the microbiome, which will hopefully lead to future insights into GC prevention and treatment.

### Supplementary Information

**Supplementary Material 1.**

**Supplementary Material 2.**

## References

[1] Bray F (2018). Global cancer statistics 2018: GLOBOCAN estimates of incidence and mortality worldwide for 36 cancers in 185 countries. CA Cancer J Clin.

[2] Vinasco K (2019). Microbial carcinogenesis: lactic acid bacteria in gastric cancer. Biochim Biophys Acta Rev Cancer.

[3] Mentis A-FA (2019). Helicobacter pylori infection and gastric cancer biology: tempering a double-edged sword. Cell Mol Life Sci.

[4] Yu G (2017). Molecular characterization of the human stomach microbiota in gastric cancer patients. Front Cell Infect Microbiol.

[5] Castaño-Rodríguez N (2017). Dysbiosis of the microbiome in gastric carcinogenesis. Sci Rep.

[6] Grochowska M (2022). The role of gut microbiota in gastrointestinal tract cancers. Arch Immunol Ther Exp.

[7] Zhang Y (2021). Gut microbiome analysis as a predictive marker for the gastric cancer patients. Appl Microbiol Biotechnol.

[8] Park JY (2022). Dysbiotic change in gastric microbiome and its functional implication in gastric carcinogenesis. Sci Rep.

[9] Wu J (2020). Fecal microbiome alteration may be a potential marker for gastric cancer. Dis Markers.

[10] Andrews S. FastQC: a quality control tool for high throughput sequence data. 2010. 2017.

[11] Bolyen E (2019). Reproducible, interactive, scalable and extensible microbiome data science using QIIME 2. Nat Biotechnol.

[12] Amir A (2017). Deblur rapidly resolves single-nucleotide community sequence patterns. MSystems.

[13] Tucker CM (2017). A guide to phylogenetic metrics for conservation, community ecology and macroecology. Biol Rev.

[14] Lozupone CA (2007). Quantitative and qualitative β diversity measures lead to different insights into factors that structure microbial communities. Appl Environ Microbiol.

[15] Liu C (2021). microeco: an R package for data mining in microbial community ecology. FEMS Microbiol Ecol.

[16] Segata N (2011). Metagenomic biomarker discovery and explanation. Genome Biol.

[17] Dhariwal A (2017). MicrobiomeAnalyst: a web-based tool for comprehensive statistical, visual and meta-analysis of microbiome data. Nucleic Acids Res.

[18] Wemheuer F (2020). Tax4Fun2: prediction of habitat-specific functional profiles and functional redundancy based on 16S rRNA gene sequences. Environ Microbiome.

[19] Buchfink B, Xie C, Huson DH (2015). Fast and sensitive protein alignment using DIAMOND. Nat Methods.

[20] Gunathilake M (2021). Association between bacteria other than Helicobacter pylori and the risk of gastric cancer. Helicobacter.

[21] Liang W (2019). Gut microbiota shifts in patients with gastric cancer in perioperative period. Medicine.

[22] Nada HG (2020). Lactobacillus acidophilus and Bifidobacterium longum exhibit antiproliferation, anti-angiogenesis of gastric and bladder cancer: impact of COX2 inhibition. PharmaNutrition.

[23] Yu D (2021). Fecal streptococcus alteration is associated with gastric cancer occurrence and liver metastasis. Mbio.

[24] Guo Y (2020). Effect of Helicobacter pylori on gastrointestinal microbiota: a population-based study in Linqu, a high-risk area of gastric cancer. Gut.

[25] Lou L (2020). Sex difference in incidence of gastric cancer: an international comparative study based on the Global Burden of Disease Study 2017. BMJ Open.

[26] Deng Y (2021). Alterations in mucosa-associated microbiota in the stomach of patients with gastric cancer. Cell Oncol.

[27] Ghazvini K, Youssefi M, Keikha M (2020). The in silico evaluation of microbial community of gastric microbiota and their role in dyspepsia in two populations from southwestern in Colombia. Gene Rep.

[28] Dingemanse C (2015). Akkermansia muciniphila and Helicobacter typhlonius modulate intestinal tumor development in mice. Carcinogenesis.

[29] Osman MA (2021). Parvimonas micra, Peptostreptococcus stomatis, Fusobacterium nucleatum and Akkermansia muciniphila as a four-bacteria biomarker panel of colorectal cancer. Sci Rep.

[30] Litvak Y (2017). Dysbiotic Proteobacteria expansion: a microbial signature of epithelial dysfunction. Curr Opin Microbiol.

[31] Madueño L (2021). Assessment of biological contribution to natural recovery of anthropized freshwater sediments from Argentina: autochthonous microbiome structure and functional prediction. Front Microbiol.

[32] Schneeberger M (2015). Akkermansia muciniphila inversely correlates with the onset of inflammation, altered adipose tissue metabolism and metabolic disorders during obesity in mice. Sci Rep.

[33] Zhang F, Du G (2012). Dysregulated lipid metabolism in cancer. World J Biol Chem.

[34] Su S-C (2021). Oral microbial dysbiosis and its performance in predicting oral cancer. Carcinogenesis.

[35] Mitsui T, Saito M, Harasawa R (2018). Salivary nitrate-nitrite conversion capacity after nitrate ingestion and incidence of Veillonella spp. in elderly individuals. J Oral Sci.

[36] Kurmi K, Haigis MC (2020). Nitrogen metabolism in cancer and immunity. Trends Cell Biol.

[37] Retnakumar RJ (2022). Gastrointestinal microbiome in the context of Helicobacter pylori infection in stomach and gastroduodenal diseases. Prog Mol Biol Transl Sci.

[38] Wen J (2021). Gastric microbiota beyond *H. pylori*: an emerging critical character in gastric carcinogenesis. Biomedicines.

[39] He C (2022). Convergent dysbiosis of gastric mucosa and fluid microbiome during stomach carcinogenesis. Gastric Cancer.

[40] Sarhadi V (2021). Gut microbiota of patients with different subtypes of gastric cancer and gastrointestinal stromal tumors. Gut Pathog.

[41] Miao Y (2022). Gut microbiota dysbiosis in the development and progression of gastric cancer. J Oncol.

[42] Gao J-J (2018). Association between gut microbiota and Helicobacter pylori-related gastric lesions in a high-risk population of gastric cancer. Front Cell Infect Microbiol.

[43] Klymiuk I (2017). The human gastric microbiome is predicated upon infection with Helicobacter pylori. Front Microbiol.

[44] Huang K (2021). Salivary microbiota for gastric cancer prediction: an exploratory study. Front Cell Infect Microbiol.

