# Supplementary material for: Gut metagenomic analysis of gastric cancer patients reveals Akkermansia, Gammaproteobacteria, and Veillonella microbiota as potential non-invasive biomarkers
Source: Genomics Inform. 2024 May 21;22:1. doi: 10.1186/s44342-024-00001-8 (PMC11184957; doi:10.1186/s44342-024-00001-8)
Supplement: Supplementary file 1 — Supplementary Material 1. [file 44342_2024_1_MOESM1_ESM.docx]

**Gut Metagenomic Analysis of Gastric Cancer Patients Reveals *Akkermansia, Gammaproteobacteria, Veillonella* Microbiota as Potential Non-invasive Biomarkers**

**Interdisciplinary Sciences: Computational Life Sciences**

Anju R. Nath, Jeyakumar Natarajan^*^

Data Mining and Text Mining Laboratory, Department of Bioinformatics, Bharathiar University, Coimbatore 641 046, India

*Corresponding author: E-mail address: n.jeyakumar@yahoo.co.in

**Supplementary Figure 1**

A)


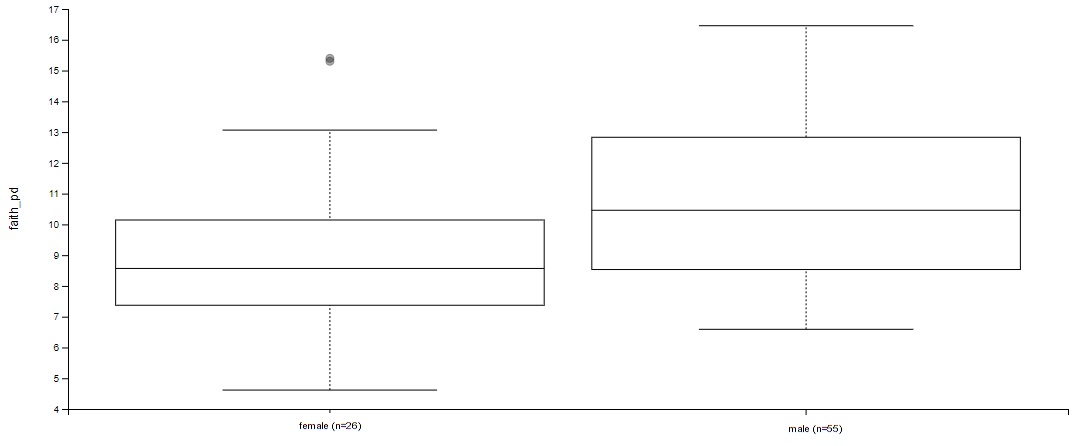


B)


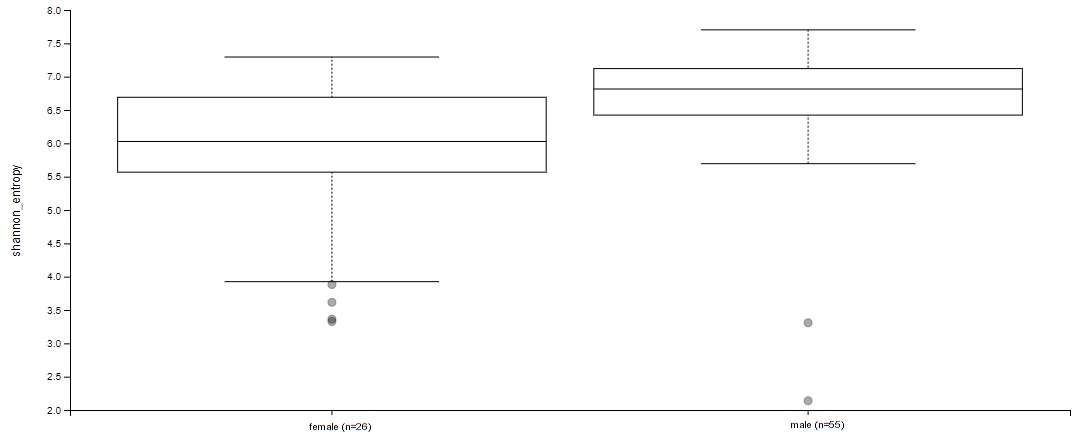


Figure 1: α diversity pair-wise values of A) Faith’s PD and B) Shannon indices for male and female with GC.

**Supplementary Figure 2**

A)
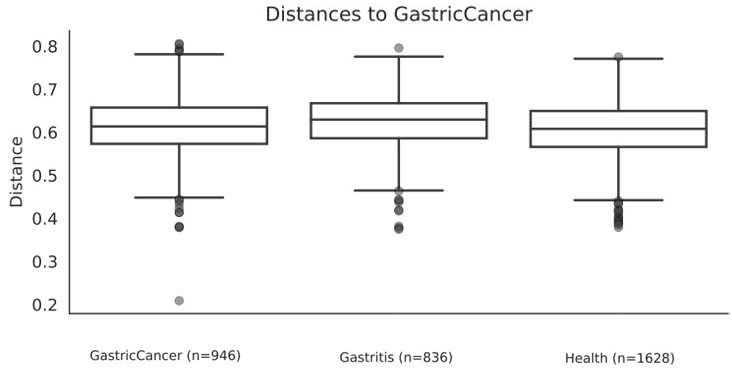


B)


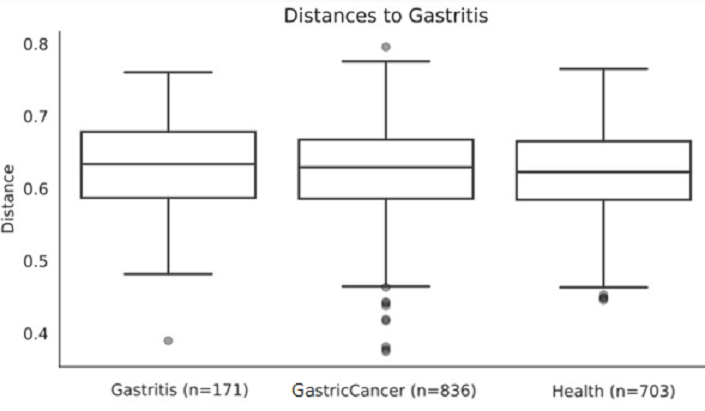


C)


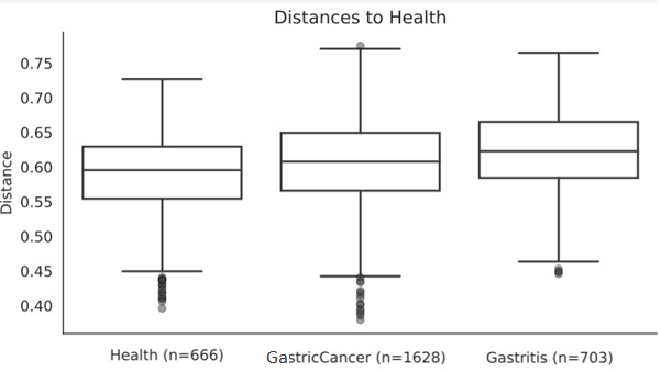


Figure 2: Box plot of unweighted UniFarc distance matrix A) Gastric Cancer B) Gastritis C) Health

**Supplementary Figure 3**

A)


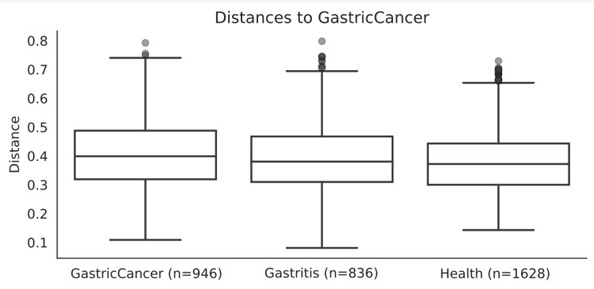


B)


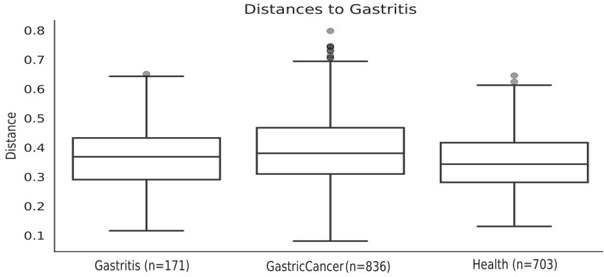


C)
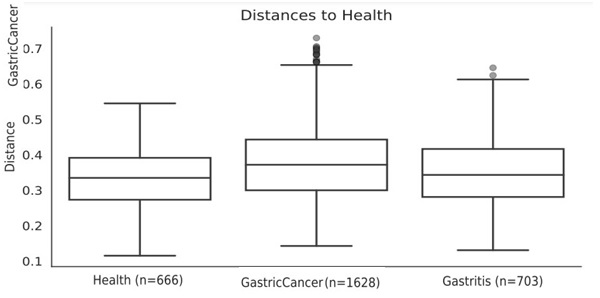


Figure 3: Box plot of weighted UniFarc distance matrix A) Gastric Cancer B) Gastritis C) Health

**Supplementary Figure 4**

**A)**


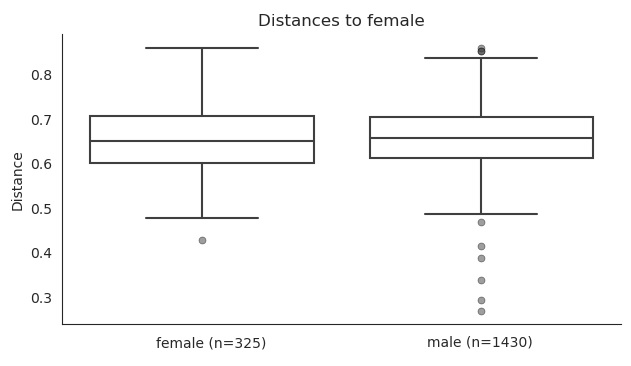


B)
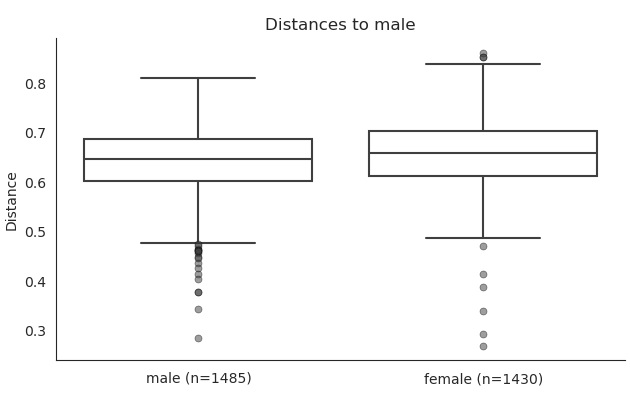


C)


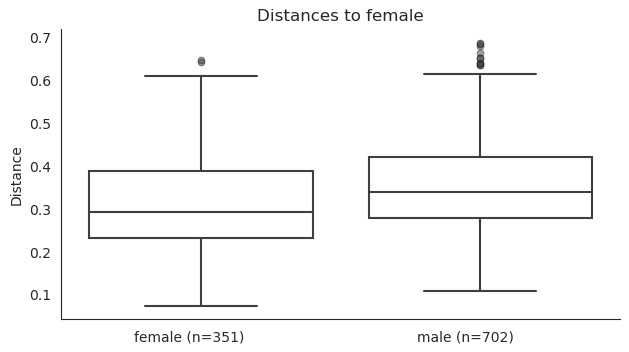


D)


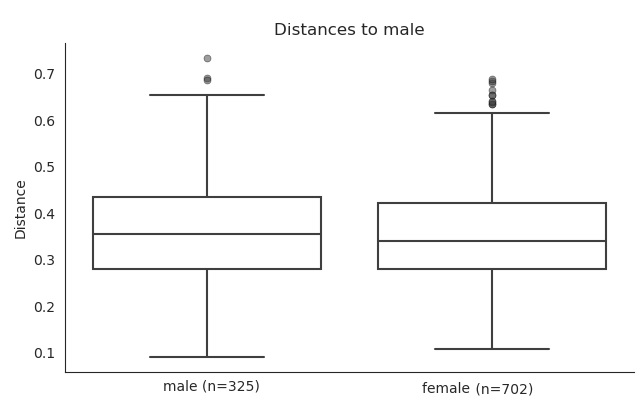


Figure 4: β diversity pair-wise values of unweighted for A) female and B) male with GC and weighted for C) female and D) male with gastritis.
